# Granuloma Annulare – A Manifestation of Primary Cutaneous Mucinous Carcinoma?

**DOI:** 10.7759/cureus.12304

**Published:** 2020-12-26

**Authors:** Buket Bagci, Albert Alhatem, Ozlem Fidan-Ozbilgin

**Affiliations:** 1 Pathology and Laboratory Medicine, St. Barnabas Hospital, Livingston, USA; 2 Pathology, Rutgers University, Newark, USA; 3 Pathology, East Orange Veteran Affairs Medical Center, East Orange, USA

**Keywords:** granuloma annulare, primary mucinous carcinoma of skin, paraneoplastic, primary cutaneous mucinous carcinoma, paraneoplastic granuloma annulare

## Abstract

The objective of this report is to present a rare case of primary cutaneous mucinous carcinoma (PCMC) manifesting with granuloma annulare (GA), and to discuss the association as a paraneoplastic phenomenon.

A 65-year-old female presented with a painless, slow-growing, cystic nodule less than 1 cm over the left lateral canthus. The clinical presentation was highly suspicious of sebaceous cyst.

The histopathologic examination revealed variable sizes of neoplastic cell clusters in a pool of abundant mucin. A focus of palisading lympho-histiocytic infiltrate surrounding a necrobiosis suggestive of granuloma annulare adjacent to the tumor is identified. Series of extensive investigations performed did not reveal any primary origin.

GA can rarely be associated with various malignant conditions. Its association and prognostic importance to these conditions are unclear. The presence with certain malignancies and the resolution of GA with the treatment of underlying malignancy are an indicator that this condition can very well be a paraneoplastic phenomenon.

## Introduction

A primary cutaneous mucinous carcinoma (PCMC) is a rare malignant adnexal entity that was first described by Lennox et al. in 1952. The incidence rate is less than 0.1 per 1 million [1]. To date, approximately 200 cases have been reported. The age of incidence is between 5th and 7th decade [2]. Men are more likely to be affected than women [3]. The entity was initially named “Eccrine Mucinous Carcinoma of the Skin”, as it was believed to originate from the eccrine glands. However, recently researchers revealed its origination from the apocrine glands as well. The most common location of occurrence is the head and neck region, although other sites such as axilla, abdominal wall, vulva, scrotum, penis, groin, and extremities have been reported [3-5].

PCMC clinically presents as a slow-growing, cystic or nodular, raised lesion that gradually develops over months to years. Because the clinical presentation of PCMC is nonspecific, the differential diagnosis is vast and it includes various benign and malignant entities, such as epidermoid cyst, pyogenic granuloma, melanoma, sebaceous cyst, sebaceous carcinoma, cystic basal cell carcinoma, neuroma, lacrimal sac tumor, hemangioma, pilomatricoma, lipoma and metastatic adenocarcinoma [6].

The current treatment for PCMC is complete excision with clear margins, as the incomplete excision leads to a local recurrence, which its rate has been estimated as 30-40% of cases [7]. The confirmed clear margins by the intra-operative frozen examinations have shown to have only 7% recurrence rate [8]. Resistance to chemotherapy and radiotherapy highlights the importance of the complete resection and close follow-up. There are currently no guidelines in place for a follow-up schedule. Poor prognosis and higher risk of recurrence have been identified in Asian population with large lesions over 1.5 cm and tumors in the trunk region.

Granuloma annulare (GA) is a self-limiting granulomatous inflammatory disease, first described by Thomas Fox in 1895. Multiple clinical variants exist, including localized, generalized, subcutaneous and perforating [9, 10]. Its association with various conditions, such as infections, metabolic diseases and disturbances, sarcoidosis, allergy, and rarely with solid organ, hematologic and lymphoid malignancies, is well known [11-18]. Although, the association of malignancies is well established in the literature, no association of PCMC with GA has ever been reported.

## Case presentation

**Figure 1:**
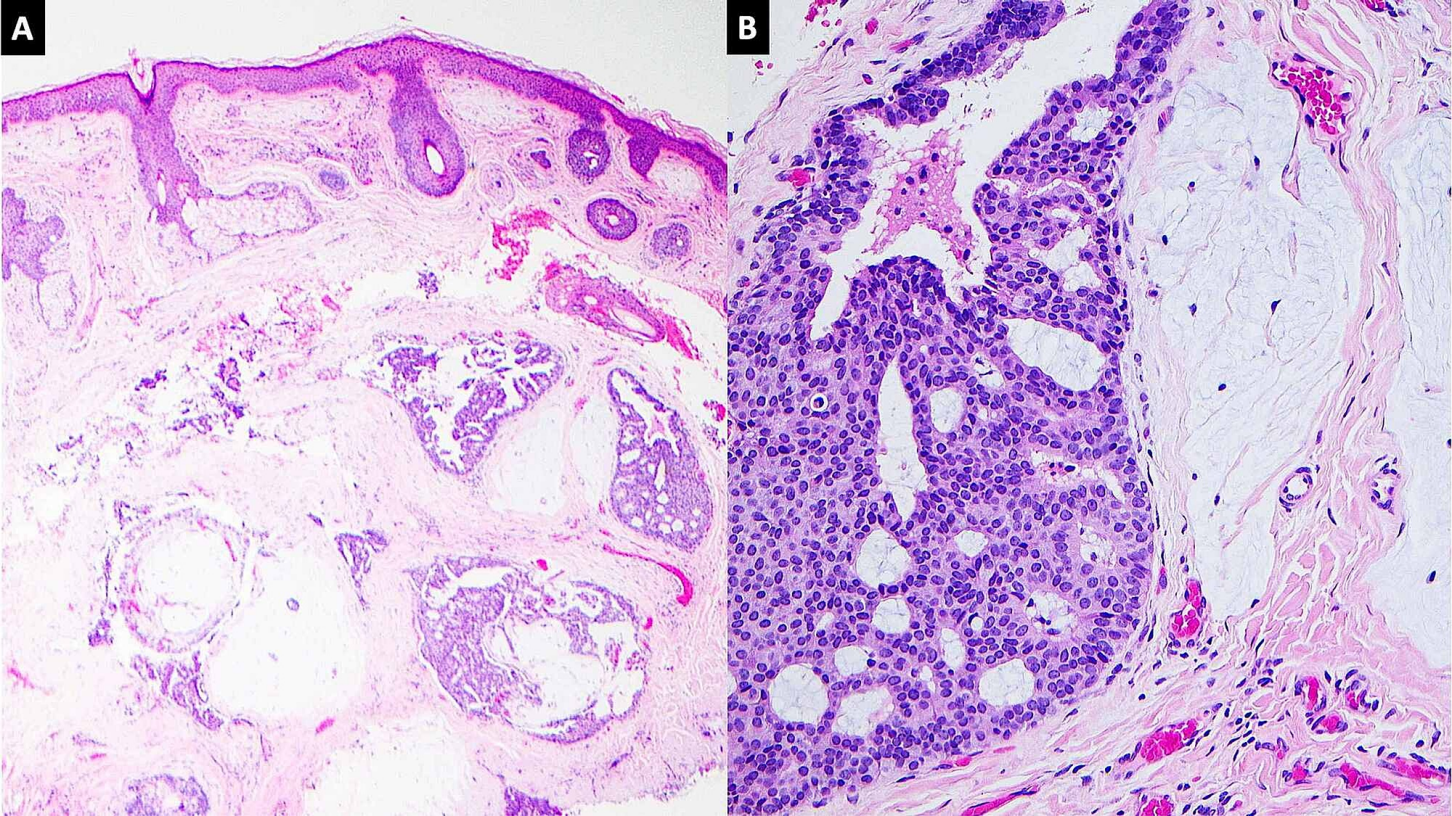
(A) Hematoxylin and eosin (H&E) staining (10x) identifying pools of mucin and clusters of neoplastic cells. (B) H&E staining identifying cribriforming cluster of neoplastic cells.

A 65-year-old woman presented with a cystic lesion over the left lateral canthus. The lesion grew slowly over the years. On examination, the lesion was brownish to yellow, cystic with an irregular surface and was measuring 0.9 cm in the largest dimension. No ulceration or oozing was identified. No pain was elicited upon palpation. The clinical appearance was consistent with sebaceous cyst. A decision to excise it was made due to aesthetic reasons. The bisection of the specimen revealed a well-circumscribed cyst, filled with homogenous yellow fluid. The routine hematoxylin and eosin stain revealed variably sized nests of dermal neoplastic cells embedded in pools of mucin. Cribriform architecture in these nests of neoplastic cells is identified (Figure 1).

**Figure 2:**
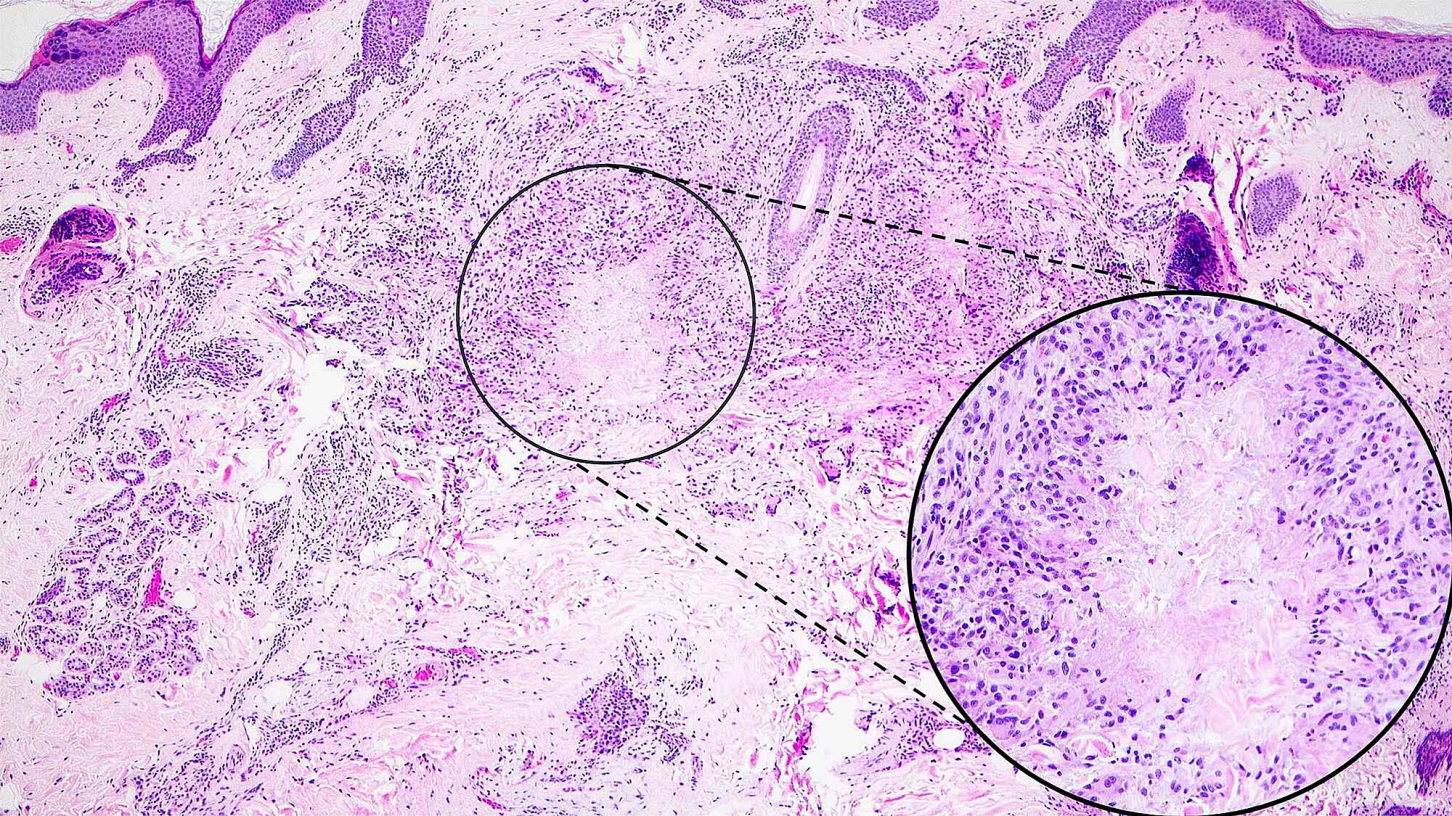
Hematoxylin and eosin (H&E) staining (10x and 40x) identifying a focus necrobiosis.

**Figure 3:**
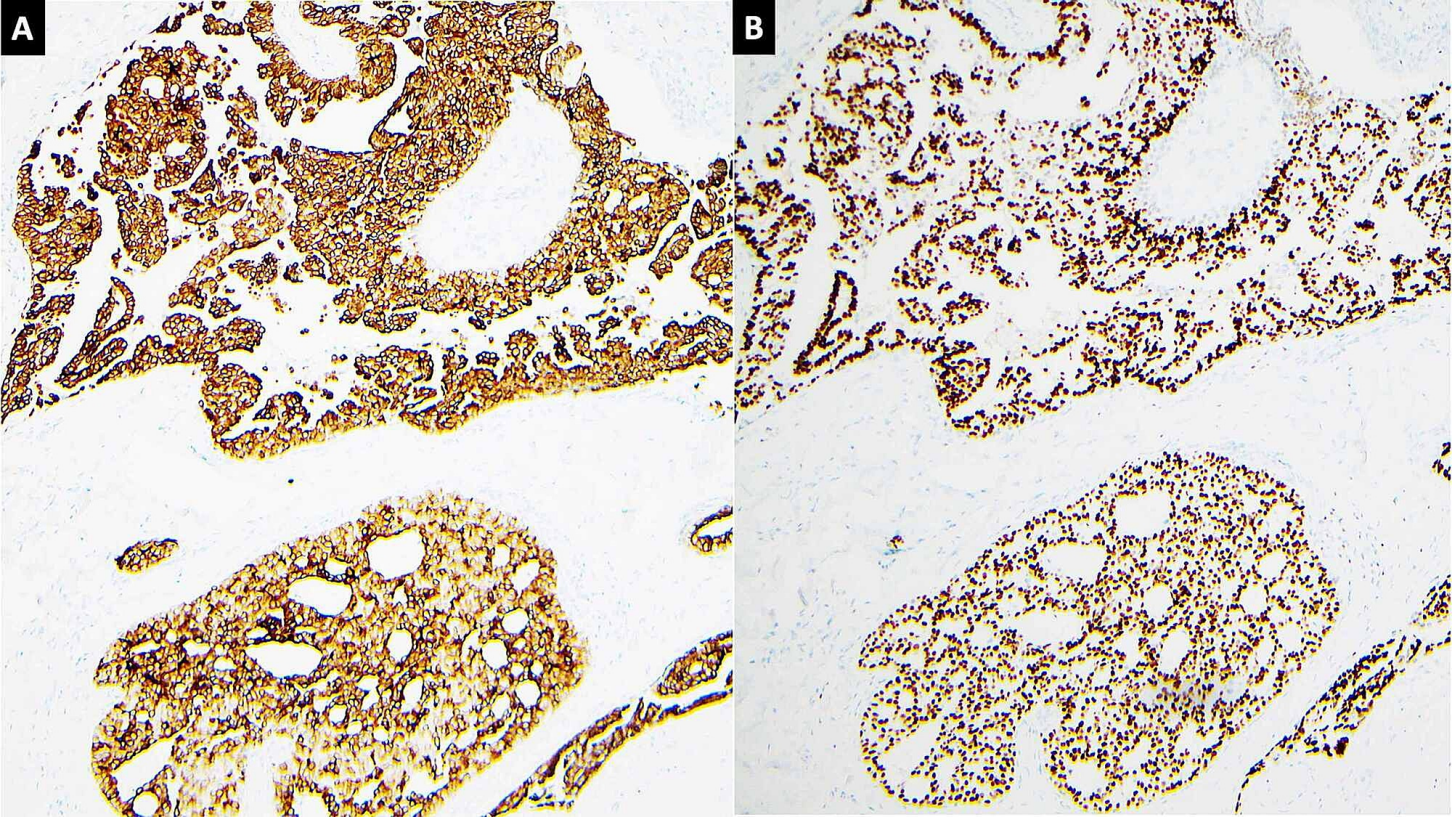
(A) CK7 staining highlights the neoplastic cells of primary cutaneous mucinous carcinoma (PCMC). (B) Gata3 staining highlights the neoplastic cells of PCMC.

The neoplastic cells were monotonous, midsized with a round to oval nuclei. No mitosis was identified. Additionally, a focus of necrobiosis surrounded with predominantly mononuclear palisading lymphohistiocytic infiltrate in the dermis less than 1 mm away from the neoplastic cells was present (Figure 2). These findings were consistent with classic granuloma annulare. Immunohistochemical (IHC) staining with cytokeratin 7, low molecular cytokeratin, and GATA3 were strongly positive in the neoplastic cells (Figure 3). However, IHC staining with CK20, CDX2 and TTF-1 was negative (not shown). Multiple levels screened failed to reveal an in-situ component. The absence of in-situ component was concerning for metastasis, which triggered numerous work-up tests to exclude it. In addition, her mammography six months prior to the lesion excision was negative. The postoperative pelvic MRI, pelvic ultrasound, abdominal CT, chest X-ray and repeated mammography showed no evidence of primary origin. In light of these findings, the diagnosis of PCMC was favored.

## Discussion

We presented a rare case of primary cutaneous mucinous carcinoma (PCMC) with granuloma annulare in 65-year-old woman. The etiology of GA is poorly understood, but recent reports have implemented the cell-mediated delayed hypersensitivity reaction [13]. It can happen anywhere in the body with extremities being the most common location. The typical lesion of GA is characterized by an annular, erythematous plaque with histopathology showing palisaded or interstitial infiltrate of lymphocytes and histiocytes with increased mucin deposition [9]. The histopathologic review showed no significant differences between paraneoplastic and classic GA [11]. The only difference is the presence of a more prominent perivascular inflammation in the paraneoplastic GA. Morphologically, our case presented with a pattern of palisaded lymphohistiocytic infiltrate (Figure 2). Minimal perivascular inflammation was observed. Additionally, when it is associated with the malignancies, they have shown to be refractory to conventional treatment of GA [11].

These cases have shown the resolution after the treatment of the underlying malignancy. The removal of the PCMC resolved the GA and during postoperative four and six months follow-up there was no evidence of recurrence. The paraneoplastic GA has shown minimal male-to-female dominance (8:7), whereas generalized GA has shown female predominance (2.2:1) [10]. The screening for malignancy in the presence of GA is a great challenge, and current guidelines are based on both age-appropriate cancer screening, patient-specific risk factors, and the clinical behavior of GA. In our case, GA adjacent to the malignancy was limited to the temporal area, and no other sites were identified.

The diagnosis of PCMC is challenging for both clinicians and pathologists. Clinically, PCMC can mimic various benign conditions. Morphologically, there is no specific finding to distinguish PCMC from metastatic mucinous carcinomas. Both entities morphologically present with large pools of mucin with monotonous neoplastic cells with round to oval nuclei. The clusters of neoplastic cells arranged in a cribriform or nesting pattern. The mitotic rate is usually low. The presence of peripheral myoepithelial cell lining suggests an in-situ component has been commonly identified in PCMC and strongly suggests skin as the primary origin. In our case, the absence of in-situ component was concerning for metastatic PCMC, however, serial preoperative, postoperative and follow-up investigations did not reveal any primary origin.

Series of IHC staining are used to identify PCMC. The examples are CK7, p63, EMA, CEA, CK20, CDX2, and GATA3 [19, 20]. There is currently no specific IHC staining for PCMC. The CK7 and p63 staining highlight the myoepithelial lining and the neoplastic cells in PCMC. However, mucinous carcinoma of breast and lung also express these markers. The mucinous carcinomas that may commonly metastasize to the skin are mucinous carcinoma of the breast, colon, and lung carcinomas. Nevertheless, the diagnosis of PCMC is rendered as a diagnosis of exclusion in the absence of primary origin.

## Conclusions

In summary, because of proximity to PCMC and resolution after the treatment of malignancy, we believe GA is one of the signs associated with PCMC. In patients presenting with a histopathologic pattern of palisaded lymphohistiocytic infiltrate with the presence of a more prominent perivascular inflammation GA, PCMC should be added to the list of malignancies to consider. However, further studies are required to establish the etiology, prognosis and management of this highly suspected paraneoplastic phenomenon.
